# Honey dressing for diabetic foot ulcers: a systematic review and meta-analysis of randomized controlled trials

**DOI:** 10.3389/fendo.2026.1759703

**Published:** 2026-03-18

**Authors:** Ling Yao, Jiezhi Dai, Shasha Mei

**Affiliations:** 1 Department of Orthopedic Surgery, The Affiliated Hospital (GROUP) of Putian University, Putian, Fujian, China; 2 Department of Orthopedic Surgery, Shanghai Sixth People’s Hospital, JiaoTong University, Shanghai, China; 3 Department of Nursing, Shanghai Sixth People’s Hospital, JiaoTong University, Shanghai, China; 4 Department of Anesthesiology, Shanghai Sixth People’s Hospital, JiaoTong University, Shanghai, China

**Keywords:** diabetic foot ulcer, honey dressing, meta-analysis, RCT - randomized controlled trial, wound healing

## Abstract

**Background:**

To systematically evaluate the efficacy and safety of honey dressing compared to conventional dressings in the treatment of DFUs.

**Methods:**

We conducted a systematic review and meta-analysis following PRISMA guidelines. PubMed, BIOSIS, EMBASE, Cochrane Central Register of Controlled Trials (CENTRAL), and Google Scholar internet were searched from inception to Jan 31, 2026 for RCTs comparing honey dressing with conventional dressings in DFU patients. Primary outcomes were complete wound healing rate and time to complete healing. Risk of bias was assessed using the Cochrane RoB 2.0 tool. The quality of evidence was graded through the Grading of Recommendations Assessment Development and Evaluations (GRADE) approach.

**Results:**

Sixteen RCTs involving 1423 participants were included. The sample sizes ranged from 23 to 348 participants. The follow-up duration varied from 4 to 24 weeks. Meta-analysis showed that honey dressing significantly improved the complete healing rate (OR 2.28, 95% CI 1.76 to 2.95) and reduced the time to complete healing (MD -4.38, 95% CI -8.06 to -0.71) compared to controls. According to the GRADE system, the overall quality of evidence for the outcomes of time to healing was rated as ‘Low’, and the quality of evidence for the outcomes of healing rate was rated as ‘moderate’.

**Conclusion:**

Honey dressing is a safe and more effective intervention for DFUs than conventional dressings, associated with significantly improved healing rates and faster healing time.

**Systematic Review Registration:**

https://www.crd.york.ac.uk/PROSPERO/view/CRD420251244450, identifier CRD420251244450.

## Introduction

Diabetic foot ulcers (DFUs) is one of the most severe and chronic complications of diabetes (1). These wounds substantially increase the risk of infection and lower extremity amputation, which are among the leading causes of diabetes-related mortality and long-term disability (2). With a global prevalence ranging from 4% to 10% (3), DFUs impose a considerable economic burden and markedly impair patients’ quality of life (4).

Wound dressing constitutes a critical element of DFU treatment. An optimal wound dressing for DFUs should provide effective hemostasis, possess anti-infection properties, and actively promote tissue repair (5). Medical-grade honey, applied typically via sterilized surgical gauze, has been specifically processed to meet physicochemical standards for clinical use (6). Evidence suggests that honey exhibits broad-spectrum antibacterial activity, aids in infection control, facilitates re-epithelialization, and reduces periwound inflammation (7). Gulati et al. conducted a prospective randomized study comparing the efficacy of honey dressing versus povidone-iodine dressing in chronic wound healing, demonstrating a significantly greater reduction in wound surface area and pain score, along with a more pronounced improvement in wound comfort score, in the honey dressing group compared to the povidone-iodine group (8). While other studies have reported similar outcomes, the available evidence remains limited in both quantity and methodological quality.

Therefore, to quantitatively evaluate the efficacy of honey dressing in the treatment of DFUs and to provide an evidence base for clinical decision-making, we conducted this meta-analysis. Building on prior findings, our study aims to systematically appraise the impact of honey dressing on DFU healing outcomes.

## Method

This review was conducted in accordance with the Preferred Reporting Items for Systematic Reviews and Meta-Analyses (PRISMA) guidelines (9). The protocol was registered at PROSPERO (Registration number: CRD420251244450).

### Inclusion and exclusion criteria

Eligibility was defined according to the PICOS (Participants, Intervention, Comparison, Outcomes, Study design) framework.

1. Participants: Adult patients (≥18 years) with a diagnosis of DFU.
2. Intervention: Topical application of any registered medical-grade honey dressing.
3. Comparison: Conventional or standard wound dressings (e.g., saline gauze, povidone-iodine, hydrocolloid, alginate, or foam dressings).
4. Outcomes: Complete wound healing rate and time to complete healing.
5. Study Design: Published and unpublished randomized controlled trials (RCTs).

Studies involving animal experiments or those designed as case reports were excluded. Additionally, studies with incomplete or non-extractable data were excluded from the analysis.

### Search strategy

A systematic search was performed in PubMed, BIOSIS, EMBASE, Cochrane Central Register of Controlled Trials (CENTRAL), and Google Scholar internet from database inception to Jan 31, 2026. The search strategy utilized a combination of keywords and MeSH terms: (‘honey’ OR ‘honey dressing’) AND (‘diabetic foot’ OR ‘diabetic wound’ OR ‘diabetic foot ulcer’) AND (‘randomized controlled trial’ OR ‘RCT’). We reviewed references from the original trials, grey literature, and review articles to identify potentially eligible articles.

### Study selection and data extraction

Two reviewers independently screened the titles, abstracts, and subsequently the full texts of potentially eligible studies. Disagreements were resolved through discussion or by a third reviewer. A pre-piloted data extraction form was used to collect details on study design, author, publication year, region, participant characteristics (age, sex, ulcer grade, and sample size), intervention protocols, comparator details, outcome measures, and results.

### Risk of bias assessment

The methodological quality of the included RCTs was assessed independently by two reviewers using the Cochrane Risk of Bias tool (RoB 2.0) for randomized trials (10), evaluating bias arising from random sequence generation, allocation concealment, blinding of participants and personnel, blinding of outcome assessment, incomplete outcome data, selective reporting, and other bias. The quality of evidence was graded through the Grading of Recommendations Assessment Development and Evaluations (GRADE) approach. The GRADE system summarized evidence quality on a 4-point scale (very low to high) (11).

### Data synthesis and analysis

Data were analyzed using Review Manager (RevMan) software version 5.0. For dichotomous outcomes, we calculated the odds ratio (OR) with 95% confidence interval (CI). For continuous outcomes, the mean difference (MD) with 95% CI was used. Statistical heterogeneity was quantified using the I² statistic and chi-square tests, with I² > 50% or P < 0.05 considered to represent substantial heterogeneity. A random-effects model was employed when significant heterogeneity was found. We further explored the probable sources of heterogeneity by subgroup analysis. To evaluate the robustness of our findings, we performed a sensitivity analysis by iteratively removing individual studies from the meta-analysis. Publication bias was assessed through visual inspection of the funnel plots for asymmetry, supplemented by Begg’s rank correlation test (12) and Egger’s linear regression test (13). The sensitivity analysis and publication bias analyses were conducted using STATA 14.1, and a two-sided p-value < 0.05 was defined as the threshold for statistical significance.

## Results

**Figure 1:**
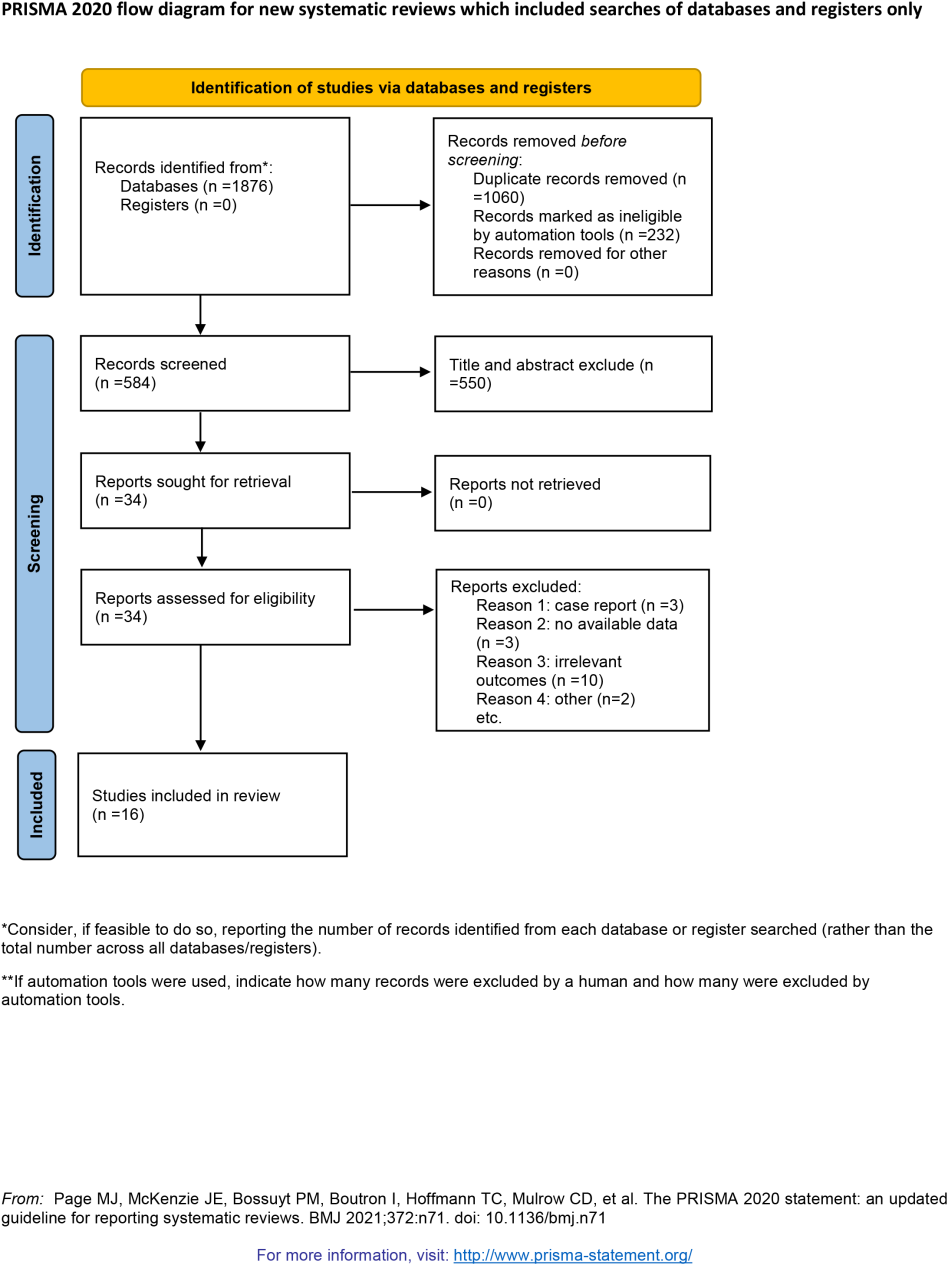
Flow diagram for study selection.

This meta-analysis included sixteen RCTs encompassing a total of 1423 participants with DFUs (14–29). The study selection process is detailed in the PRISMA flow diagram (**Figure 1**). The characteristics of the included studies are summarized in **Table 1**. The sample sizes ranged from 23 to 348 participants. The control interventions encompassed a variety of conventional dressings, including povidone-iodine gauze, saline-soaked gauze, silver dressing, paraffin tulle dressing, alginate and fucidin ointment or cream. Geographically, the studies originated from ten countries/regions, with the majority conducted in India (n=4), Pakistan (n=3), and Saudi Arabia (n=3); the remainder comprised single studies from Hong Kong, Greece, Malaysia, Egypt, Iran, and Nigeria. The follow-up duration varied from 4 to 24 weeks.

**Table 1:** The characteristics of the included studies.

Study	Design	Country	Ulcer grade	Mean age (years)	No.		Intervention		Outcome	Follow-up
					TG	CG	TG	CG		
Shukrimi 2008	RCT	Malaysia	W: 2	52.1	15	15	Honey:	Povidone iodine	2	NR
Jan 2012	RCT	Pakistan	W: 1-4	56	50	50	Honey	Povidone iodine	1	10 weeks
Eldeen 2012	RCT	Egypt	W: 2	37.9	20	20	Honey	Alginate	1,2	16 weeks
Saeed 2013	RCT	Saudi Arabia	W: 2-4	55.5	31	27	Honey	Povidone iodine	1	24 weeks
Kamaratos 2014	RCT	Greece	W: 1,2	56.5	32	31	Honey	Saline	1,2	16 weeks
Siavash 2015	RCT	Iran	T: 1,2	60.3	32	32	Honey	Placebo	1,2	12 weeks
Imran 2015	RCT	Pakistan	W: 1,2	54	179	169	Honey	Saline	1,2	16 weeks
Agarwal 2015	RCT	India	W: 1,2	52.4	18	18	Honey	Povidone iodine	2	NR
Hussain 2016	RCT	Pakistan	NR	NR	160	160	Honey	Povidone iodine	1	NR
Tsang 2017	RCT	Hong Kong, China	T: 0,1,2	65.9	10	10	Honey	Paraffin tulle	1	12 weeks
Suryaprakash 2018	RCT	India	NR	NR	6	17	Honey	Povidone iodine	2	NR
Jaiswal 2018	RCT	India	W: 2	NR	41	41	Honey	Povidone iodine	1	NR
Saeed 2019	RCT	Saudi Arabia	W: 2-4	68.4	36	35	Honey	Silver hydrogel	2	Until healed
Kateel 2023	RCT	India	W: 1,2	60	39	39	Honey	Povidone iodine	1	6 weeks
Iwunze 2024	RCT	Nigeria	W: 2	55.2	15	15	Honey	Povidone iodine	1	4 weeks
Searan 2024	RCT	Saudi Arabia	W: 2	59.64	30	30	Honey	Fucidin ointment or cream	2	12 weeks

TG, Treatment group; CG, Control group; W, Wagner Classification; T, University of Texas classification; NR, Not reported; 1. healing rate, 2. Time to complete healing.

**Figure 2:**
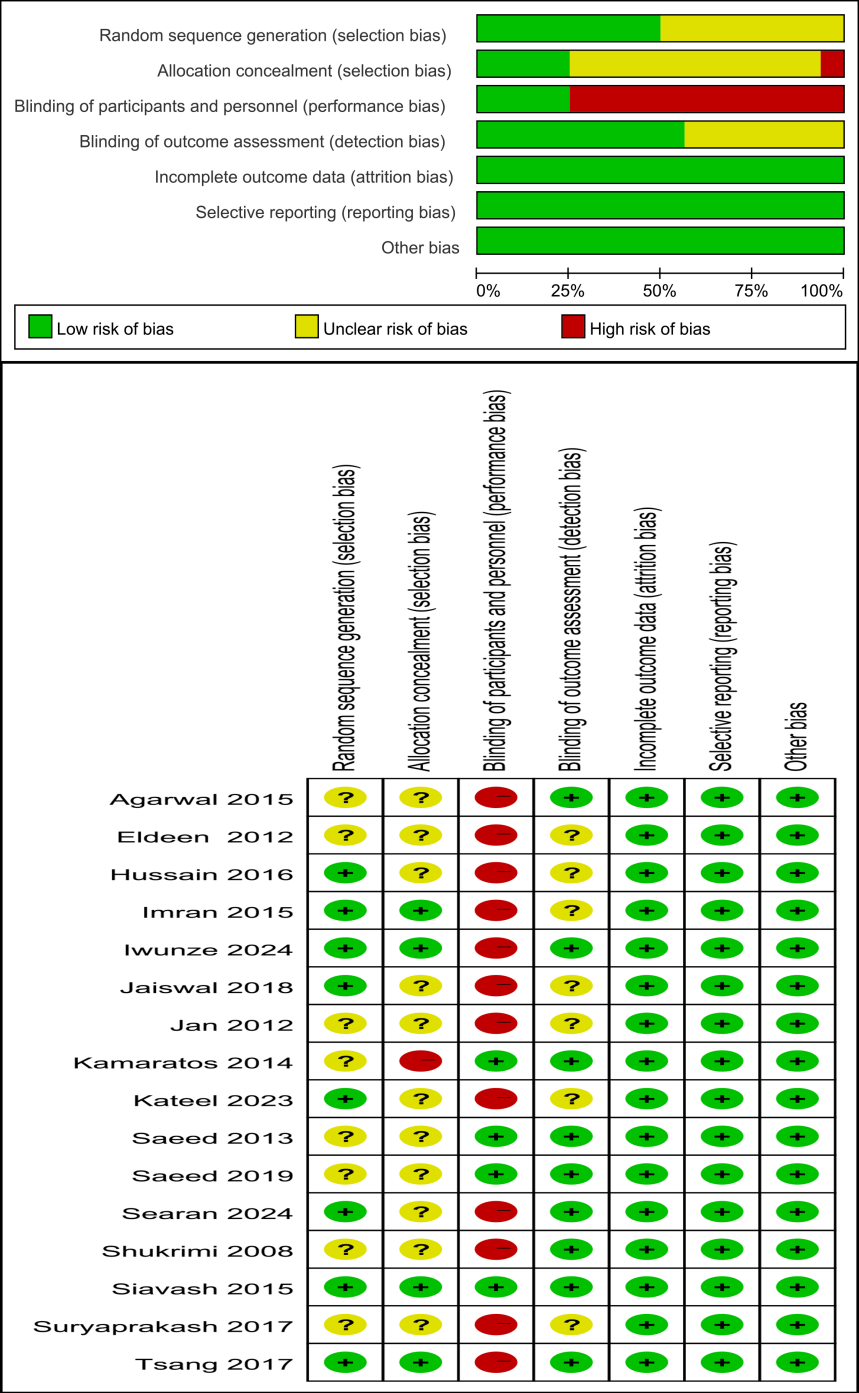
Quality evaluation of the included studies.

The risk of bias assessment is presented in **Figure 2**. In the random sequence generation domain, eight studies had an unclear risk of bias. In the allocation concealment domain, eleven studies had an unclear risk of bias, and one study had a high risk of bias. In the blinding of participants and personnel domain, twelve studies had a high risk of bias. In the blinding of outcome assessment domain, seven studies had an unclear risk of bias. In the incomplete outcome data, selective reporting, and other bias, all studies were at a low risk of bias. According to the GRADE system, the overall certainty of evidence for the outcomes of time to healing was rated as ‘Low’, owing to methodological limitations in risk of bias and substantial heterogeneity. In contrast, the certainty of evidence for the healing rate was rated as ‘moderate’. A summary of these findings is presented in **Table 2**.

**Table 2:** Grading of Recommendations Assessment, Development and Evaluation (GRADE) assessment of overall cert.

Outcomes	No. of studies	Risk of bias	Inconsistency	Indirectness	Imprecision	Publication bias	Quality of evidence
healing rate	11	Serious	Not serious	Not serious	Not serious	Not serious	⨁⨁⨁◯ Moderate
Time to healing	9	Serious	Serious	Not serious	Not Serious	Not serious	⨁⨁◯◯ Low

**Figure 3:**
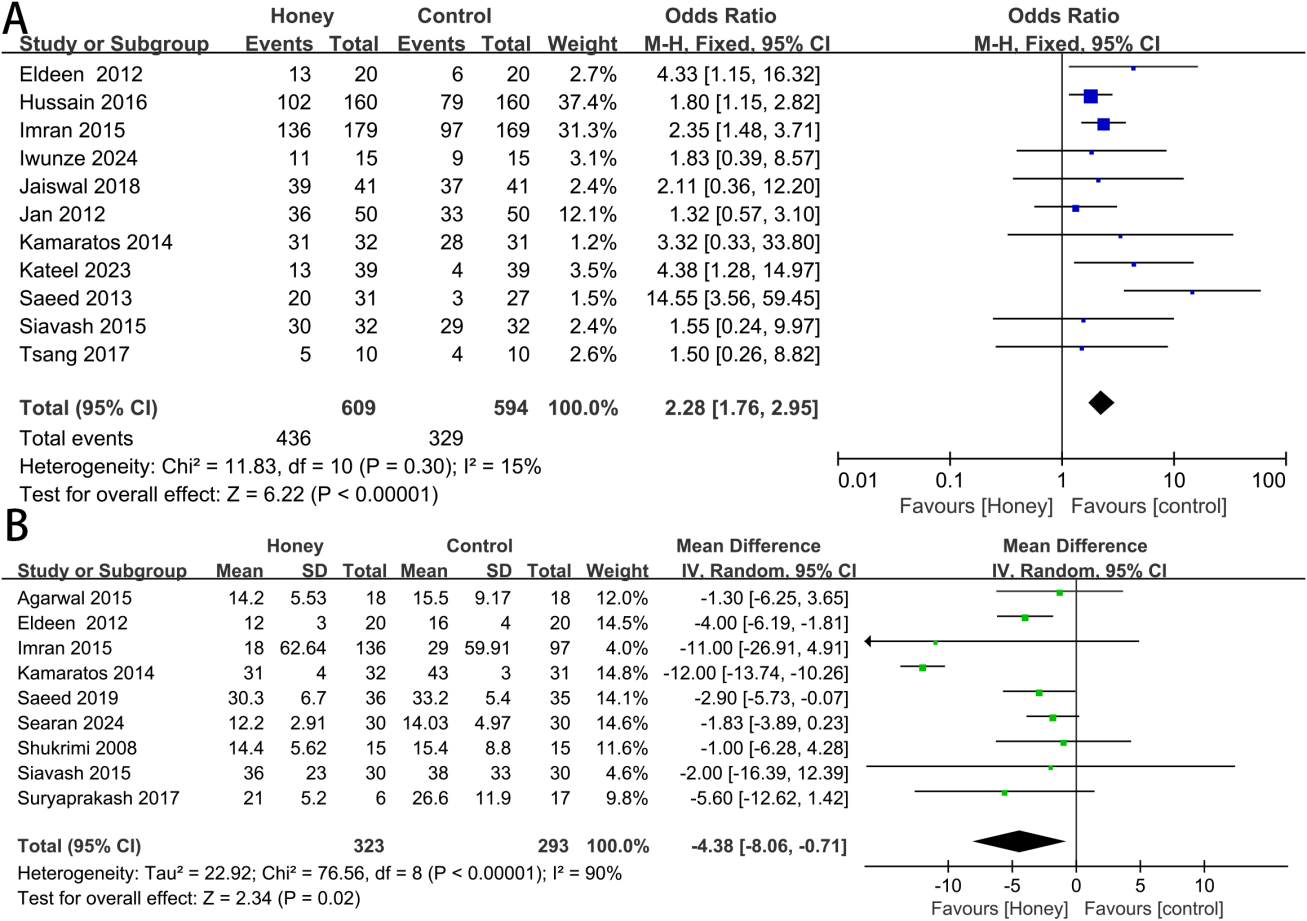
Meta-analysis of healing rate **(A)** and time to complete healing **(B)**.

**Figure 4:**
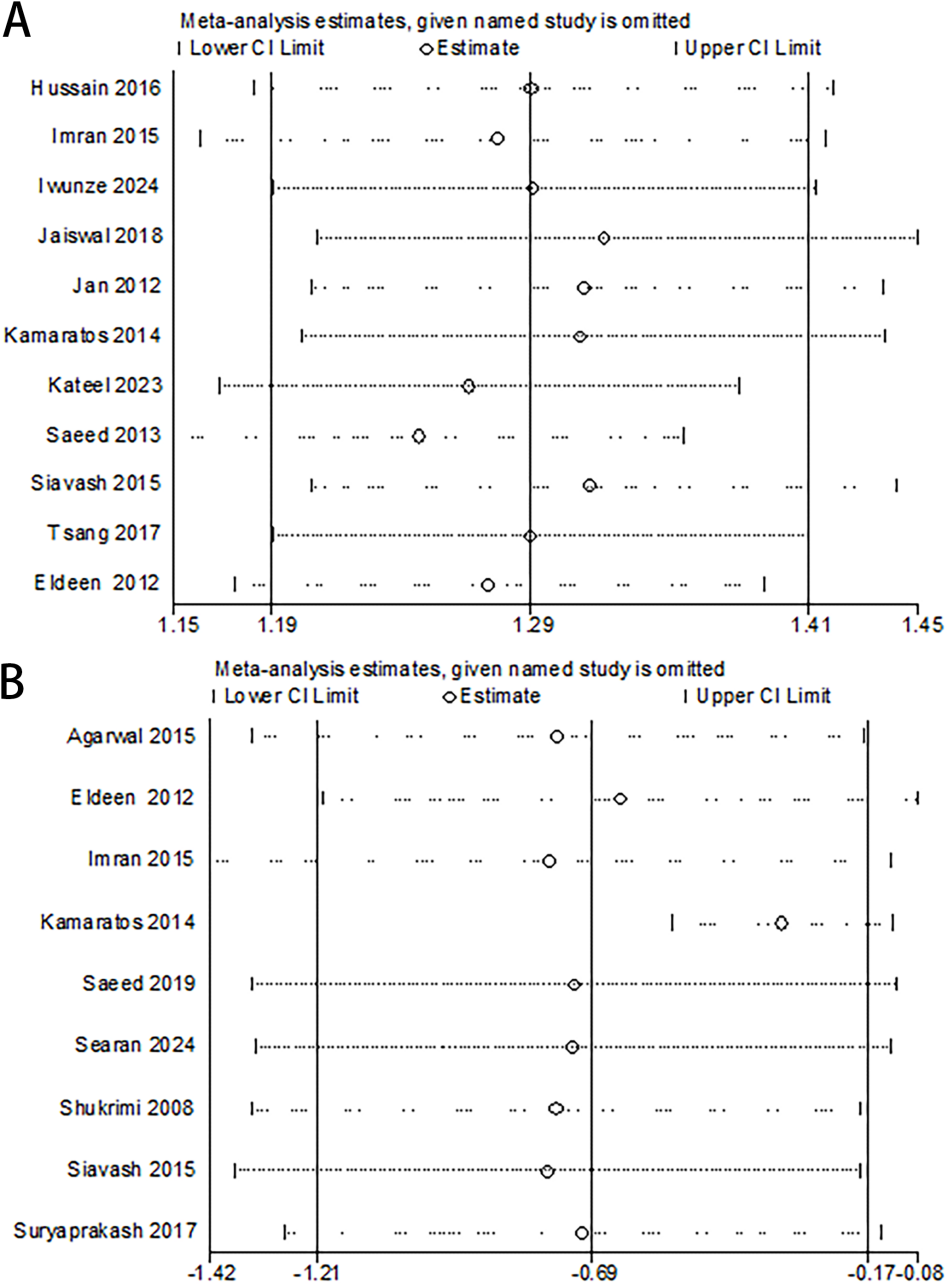
Sensitivity analysis of the meta-analysis. **(A)** Healing rate: no single study exerted a disproportionate influence on the overall effect, confirming the robustness of the primary analysis. **(B)** Time to complete healing: the study by Kamaratos et al. was identified as the primary source of heterogeneity.

Complete Healing Rate: eleven studies reported on the complete healing rate. There was no significant statistical heterogeneity between studies (P = 0.30; I² = 15%), and a fixed-effects model was used. Meta-analysis demonstrated that honey group significantly increased the likelihood of complete healing compared to control group (OR 2.28, 95% CI 1.76 to 2.95, P < 0.00001) (**Figure 3A**). According to the sensitivity analysis, there were no significant changes in the overall pooled results, indicating that the results of this study were stable and reliable (**Figure 4A**).

Time to Complete Healing: a pooled analysis of nine studies showed that honey group significantly reduced the time to complete healing (MD -4.38, 95% CI -8.06 to -0.71, P = 0.02) (**Figure 3B**). However, considerable heterogeneity was observed among these studies (P < 0.00001; I² = 90%). We conducted subgroup analyses to explore potential sources of heterogeneity (**Table 3**). The covariates included ulcer grade (Group 1: both Wagner and University of Texas classifications below grade 2; Group 2: either classification indicating grade 4; Group 3: grade not reported) and comparator dressing type (Group 1: antimicrobial dressings, e.g., povidone-iodine, silver hydrogel, fusidic acid ointment or cream; Group 2: inert dressings, e.g., saline, alginate, placebo). As the test for subgroup differences was not statistically significant, the subgroup analyses revealed that neither ulcer grade (P = 0.71) nor comparator dressing type (P = 0.11) significantly contributed to the heterogeneity in complete healing time. We performed a sensitivity analysis to detect whether one trial significantly influenced the outcomes or greatly contributed to the heterogeneity (**Figure 4B**). Notably, the study by Kamaratos et al. (25) was identified as the primary source of heterogeneity. Its subsequent exclusion yielded a more precise and homogeneous effect estimate (MD -2.79, 95% CI -4.01 to -1.57, P = 0.73; I² = 0%).

**Table 3:** Subgroup analysis relating to healing time.

Group	No.	P _heterogeneity_	I^2^	MD	95%CI	P	P _interaction_
Total	9	<0.00001	90%	-4.38	-8.06 to -0.71	0.02	
Ulcer grade							
Group 1	7	<0.00001	92%	-4.49	-9.06 to 0.08	0.05	0.71
Group 2	1	–	–	-2.90	-5.73 to -0.07	0.04	
Group 3	1	–	–	-5.60	-12.62 to 1.42	0.12	
Comparator dressings							
Group 1	5	0.81	0%	-2.18	-3.66 to -0.70	0.004	0.11
Group 2	4	<0.00001	91%	-7.57	-14.08 to -1.06	0.02	

**Figure 5:**
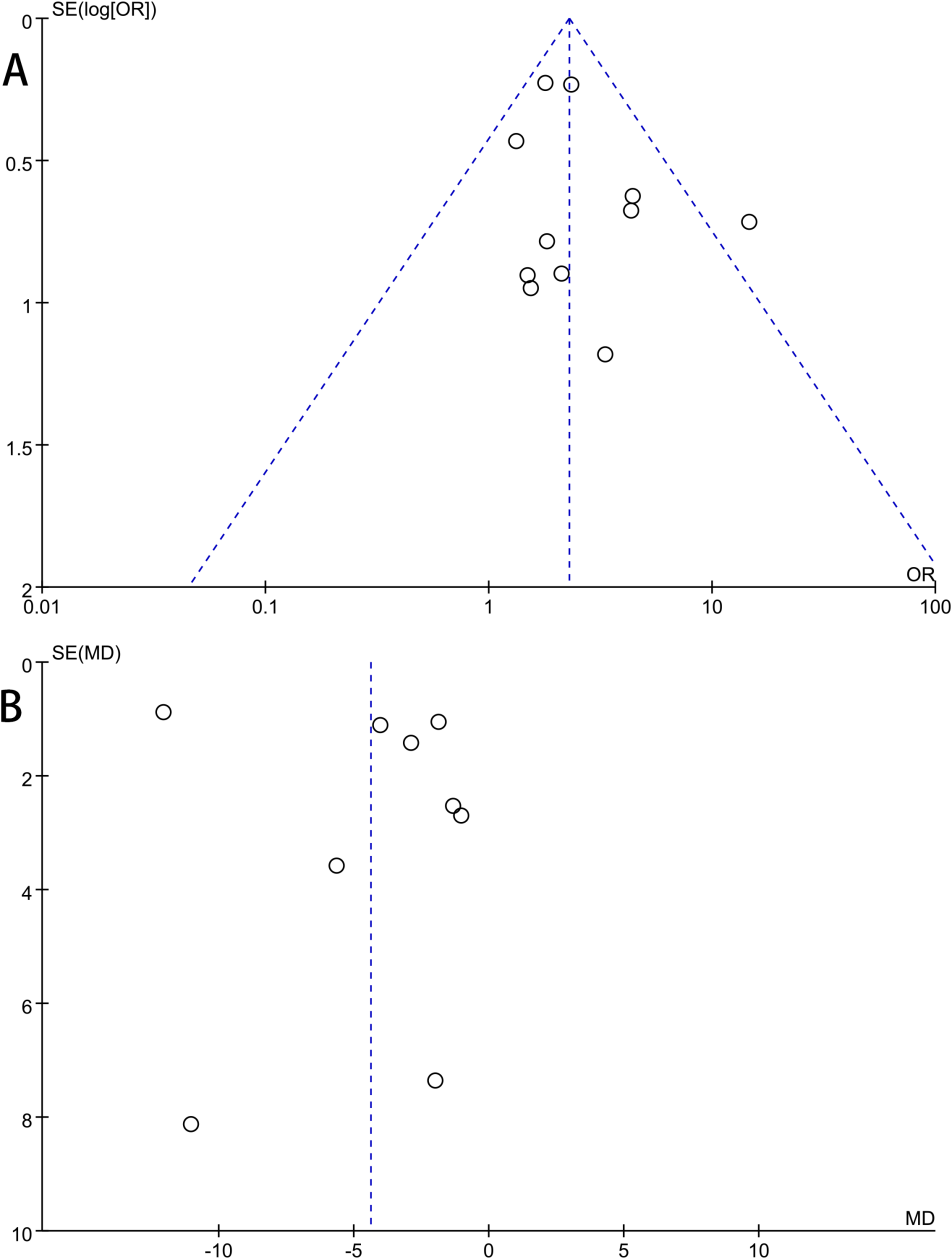
Publication bias analysis of the meta-analysis. Each dot represents an individual study. The funnel plot appeared symmetric upon visual inspection, suggesting no significant publication bias. **(A)** Healing rate; **(B)** Time to complete healing.

Publication bias was evaluated for the primary outcomes of ulcer healing rate and time to complete healing using funnel plots, Begg’s test, and Egger’s test. The funnel plots revealed no substantial asymmetry, indicating a low likelihood of substantial publication bias for the outcomes of healing rate (**Figure 5A**) and time to complete healing (**Figure 5B**). Statistical findings from Begg’s and Egger’s tests corroborated this visual assessment (Healing rate: Egger’s P = 0.327, Begg’s P = 0.876; Time to healing: Egger’s P = 0.185, Begg’s P = 0.118).

## Discussion

This systematic review and meta-analysis of sixteen RCTs provides compelling evidence that honey dressing is superior to conventional dressings for the management of DFUs. Our findings demonstrate that its application is associated with a significantly higher healing rate and shorter time to complete wound healing compared to control groups.

The superior efficacy of honey dressings stems from their multifaceted mechanisms of action. Unlike conventional dressings that serve as passive barriers, honey establishes a proactive healing microenvironment. Its high osmolarity induces bacterial dehydration, thereby inhibiting proliferation (30), while concurrently drawing exudate away from the wound bed to reduce edema and promote autolytic debridement. Furthermore, honey releases hydrogen peroxide in a sustained, low-dose manner, exerting broad-spectrum antimicrobial activity without cytotoxic effects on nascent tissue (31). This property is particularly critical for DFUs, which are highly vulnerable to infection and biofilm formation. The acidic pH of honey helps lower the alkaline environment typical of chronic wounds, creating a favorable milieu for fibroblast function and oxygen release (32). Moreover, antioxidants contained in honey-including flavonoids, monophenolics, polyphenolics, and vitamin C-reduce reactive oxygen species (ROS) and ferric anions, inhibit lipid peroxidation, and ultimately reduce oxidative stress and protect against cellular damage (33). The anti-inflammatory and antioxidant properties further mitigate persistent inflammation, which is a key barrier to healing in diabetic wounds. Collectively, these actions effectively control wound bioburden and likely underlie the observed reduction in healing time by facilitating unimpeded progression of the healing cascade. Given the low cost and wide availability of medical-grade honey compared to some advanced modern dressings, it is plausible that honey dressings could offer a cost-effective alternative, particularly in resource-limited settings.

A prior systematic review by Tang et al. evaluated honey for chronic wounds and concluded that the topical application of honey was an effective therapeutic approach for managing chronic wounds (34). However, it did not perform a subgroup analysis specific to DFUs. Furthermore, two earlier meta-analyses focusing on DFUs were limited by small sample sizes and incorporated a mixture of RCTs and non-RCTs (35, 36). This scarcity of robust evidence may reflect the historically limited application of honey dressing in DFU care. Therefore, no consensus has been established regarding the superiority of honey dressing over other dressing types for DFU management. The present meta-analysis was designed to address this gap by synthesizing evidence exclusively from RCTs.

Despite the overall pooled results suggesting a potential benefit of honey dressings, these findings must be interpreted in the context of substantial clinical heterogeneity among the included randomized controlled trials. Variations existed in ulcer classification systems (Wagner vs. University of Texas), ulcer grades (ranging from grade 0 to grade 4), comparator dressings (ranging from saline gauze to other advanced dressings), and follow-up durations (4 weeks to “until healed”), which may significantly influence the pooled effect estimates, particularly for the outcome of time to complete healing. First, the severity of the diabetic foot ulcers at baseline varied considerably, with studies employing different classification systems (Wagner vs. University of Texas) and including ulcer grades ranging from superficial (grade 0) to severe (grade 4). Given that baseline ulcer severity is a primary prognostic factor for healing, this variation likely contributes to the wide confidence intervals and statistical heterogeneity observed in our meta-analysis. Second, the diversity in comparator dressings, which range from inert dressings (e.g., saline gauze or paraffin tulle) to active antimicrobial agents (e.g., silver dressings or povidone-iodine), introduces another layer of complexity. The pooling of such diverse comparators may obscure these differential effects. Third, the inconsistency in follow-up durations, from as short as 4 weeks to an open-ended ‘until healed’, introduces potential bias in time-to-event analyses. Studies with fixed short-term follow-up may censor patients who would have healed later, potentially underestimating the true healing rate.

Although a random-effects model was employed to account for this heterogeneity, it may still affect the generalizability of our findings. Specifically, sensitivity analysis identified the study by Kamaratos et al. as a major source of heterogeneity; its exclusion yielded a more precise, homogeneous, and clinically interpretable estimate (I² statistic decreased from 90% to 0%). We further conducted subgroup analyses to explore potential sources of heterogeneity. The subgroup analyses revealed that neither ulcer grade nor comparator dressing type significantly contributed to the heterogeneity in complete healing time. However, due to the limited number of studies and the inability to perform multivariable meta-regression (owing to incomplete reporting of ulcer severity and follow-up durations across studies, as noted in **Table 1**), these findings should be considered hypothesis-generating. Further trials with standardized comparators are needed to confirm these observations.

Several limitations of both the primary studies and this review itself must be acknowledged. Our confidence in the evidence is constrained by incomplete reporting in several of the primary studies. As indicated in **Table 1**, key demographic and clinical variables including mean age, ulcer grade, and follow-up durations were listed as “NR” in multiple trials. First, it increases the risk of bias, as it prevented us from conducting planned subgroup analyses or meta-regression to explore whether patient characteristics modified the treatment effect. Second, it introduces an element of indirectness; because we cannot confirm that the unreported patient populations in some trials match our target population, the direct applicability of their results is uncertain. Consequently, this incomplete reporting contributes to a downgrading of the overall certainty of evidence according to GRADE criteria. Furthermore, publication bias, though not definitively detected by funnel plot or statistical tests, remains a potential threat in any meta-analysis, as small studies with null results are less likely to be published. Finally, while the findings of this review are promising, the limitations in the primary evidence, including substantial clinical heterogeneity, variable study quality, and the absence of formal health economic evaluations, preclude definitive recommendations for widespread clinical adoption. Any implications regarding cost-effectiveness or broad implementation should be regarded as tentative and hypothesis-generating, pending confirmation by future well-designed trials. .

Based on the limitations identified, we recommend several directions for future research. There is a need for larger, multi-center RCTs with robust blinding procedures where possible, and standardized protocols for honey type and application frequency. Future studies should directly compare honey with other established advanced wound dressings (e.g., silver or iodine) to establish its relative efficacy and cost-effectiveness. In addition, future trials should incorporate patient-reported outcomes, such as quality of life, pain during dressing changes, and acceptability of the treatment, to provide a more holistic assessment of its value.

In conclusion, this systematic review and meta-analysis provides compelling evidence that honey dressings can enhance the healing of DFUs. Its application is associated with a significantly higher healing rate and shorter time to complete wound healing compared to control groups. While honey dressings appear to be a promising option for diabetic foot ulcers, these findings are based on moderate to low certainty evidence. Clinicians should consider this uncertainty in shared decision-making with patients, and recognize that future high-quality research may alter current estimates of treatment effect.

## Data Availability

The raw data supporting the conclusions of this article will be made available by the authors, without undue reservation.
